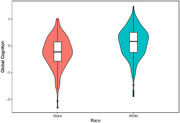# Racial Differences in Midlife Cognitive Function: Implications for AD/ADRD Risk

**DOI:** 10.1002/alz70860_107296

**Published:** 2025-12-23

**Authors:** Kumar B Rajan, Robert S. Wilson, Jerenda Bond, Lisa L. Barnes, Denis A Evans

**Affiliations:** ^1^ Rush Institute for Healthy Aging, Chicago, IL, USA; ^2^ Rush Alzheimer's Disease Center, Rush University Medical Center, Chicago, IL, USA

## Abstract

**Background:**

While racial disparities in late‐life cognitive function and Alzheimer's disease (AD) risk are well‐documented, little is known about cognitive differences between Black and White adults in midlife—a critical period for early AD‐related changes. Understanding these disparities can provide insights into cognitive aging and future dementia risk. This study examines racial differences in global cognition and domain‐specific cognitive performance among middle‐aged adults.

**Methods:**

Participants were drawn from the Parent‐Offspring Resilience and Cognitive Health (PORCH) Study, with parental data linked to the Chicago Health and Aging Project (CHAP). A standardized global cognitive function score was created from assessments of immediate and delayed logical memory, digit span (forward and backward), category fluency (animals, fruits, and vegetables), and the Mini‐Mental State Examination (MMSE). Hierarchical regression models examined racial differences in cognitive function, adjusting for family structure and demographics.

**Results:**

The study included 598 midlife adults (mean age = 56.7 ± 5.0 years, 57% female, 34% Black). Although both Black and White participants averaged a college degree (Black: 16.1 ± 2.9 years of education; White: 16.6 ± 2.3 years), Black participants had significantly lower global cognitive function scores (difference (Δ) = ‐0.35; 95% CI: ‐0.44, ‐0.25; *p* < 0.001). Differences in individual tests of cognition were also observed, with lower performance in memory (Δ = ‐0.54; *p* < 0.001), perceptual speed (Δ = ‐0.30; *p* < 0.001), and category fluency (Δ = ‐0.40; *p* < 0.001) among Black than White participants.

**Conclusions:**

Midlife cognitive disparities between Black and White adults mirror those seen in later life, suggesting that racial differences in AD/ADRD risk may emerge decades before diagnosis. These findings highlight the critical need to investigate systemic factors underlying cognitive inequities, which may inform early intervention strategies to mitigate AD risk in diverse populations.